# Effect of spinal manipulation on sensorimotor functions in back pain patients: study protocol for a randomised controlled trial

**DOI:** 10.1186/1745-6215-12-161

**Published:** 2011-06-28

**Authors:** David G Wilder, Robert D Vining, Katherine A Pohlman, William C Meeker, Ting Xia, James W DeVocht, R Maruti Gudavalli, Cynthia R Long, Edward F Owens, Christine M Goertz

**Affiliations:** 1 University of Iowa, Iowa City, IA, USA; 2 Palmer Center for Chiropractic Research, Davenport, IA, USA; 3 Palmer College of Chiropractic, San Jose, CA, USA; 4 Chiropractor/Independent Researcher, Minneapolis, MN, USA

## Abstract

**Background:**

Low back pain (LBP) is a recognized public health problem, impacting up to 80% of US adults at some point in their lives. Patients with LBP are utilizing integrative health care such as spinal manipulation (SM). SM is the therapeutic application of a load to specific body tissues or structures and can be divided into two broad categories: SM with a high-velocity low-amplitude load, or an impulse "thrust", (HVLA-SM) and SM with a low-velocity variable-amplitude load (LVVA-SM). There is evidence that sensorimotor function in people with LBP is altered. This study evaluates the sensorimotor function in the lumbopelvic region, as measured by postural sway, response to sudden load and repositioning accuracy, following SM to the lumbar and pelvic region when compared to a sham treatment.

**Methods/Design:**

A total of 219 participants with acute, subacute or chronic low back pain are being recruited from the Quad Cities area located in Iowa and Illinois. They are allocated through a minimization algorithm in a 1:1:1 ratio to receive either 13 HVLA-SM treatments over 6 weeks, 13 LVVA-SM treatments over 6 weeks or 2 weeks of a sham treatment followed by 4 weeks of full spine "doctor's choice" SM. Sensorimotor function tests are performed before and immediately after treatment at baseline, week 2 and week 6. Self-report outcome assessments are also collected. The primary aims of this study are to 1) determine immediate pre to post changes in sensorimotor function as measured by postural sway following delivery of a single HVLA-SM or LVVA-SM treatment when compared to a sham treatment and 2) to determine changes from baseline to 2 weeks (4 treatments) of HVLA-SM or LVVA-SM compared to a sham treatment. Secondary aims include changes in response to sudden loads and lumbar repositioning accuracy at these endpoints, estimating sensorimotor function in the SM groups after 6 weeks of treatment, and exploring if changes in sensorimotor function are associated with changes in self-report outcome assessments.

**Discussion:**

This study may provide clues to the sensorimotor mechanisms that explain observed functional deficits associated with LBP, as well as the mechanism of action of SM.

**Trial registration:**

This trial is registered in ClinicalTrials.gov, with the ID number of NCT00830596, registered on January 27, 2009. The first participant was allocated on 30 January 2009 and the final participant was allocated on 17 March 2011.

## Background

Low back pain (LBP) is well recognized as a public health problem, impacting up to 80% of US adults at some point in their lives [1]. Estimates of the point prevalence vary from 12-33% and lifetime prevalence vary from 11-84% [2]. The pathophysiology of patients with LBP is not well understood. An estimated 90% of LBP in clinical practice is labeled "idiopathic" [1], meaning that the mechanism is unclear. Given how little we know about the underlying causes of LBP, it is not surprising that a gold standard treatment does not currently exist. A recent survey to determine health care utilization patterns in patients with chronic LBP, found this population demonstrated an average of 21 visits annually to just under three (2.7) different provider types [3]. In conclusion, the authors state that 1) there is a high utilization of health care for chronic LBP patients, including a high rate of advanced imaging, narcotic prescription and physical therapy; 2) most of the tests and treatments performed did not meet the criteria of evidence-based practice; and 3) there is an over-utilization of treatment types. In addition to conventional medical treatments such as analgesics and physical therapy, LBP patients are also utilizing integrative health care such as spinal manipulation (SM) delivered by a doctor of chiropractic [4].

There is an increasing body of evidence suggesting that SM provides important benefit to patients with LBP [5,6]. Not much is known, however, about the mechanism of action of these treatments. This study looks closely at the biomechanical and neural effects of SM treatment.

In its broadest definition, SM involves the therapeutic application of a load (i.e. force) to specific body tissues or structures (usually vertebral joints). There are many variations of SM in terms of their velocity, amplitude, loading frequency, choice of lever, location and direction of load, and treatment frequency [7,8]. Based on SM force-time profiles, they can be divided into two broad categories: SM with a high-velocity low-amplitude load, or an impulse "thrust", to body tissues (HVLA-SM) and SM with a low-velocity variable-amplitude load (LVVA-SM) [8-12]. LVVA-SM is often referred to as "mobilization [13]," where HVLA-SM is referred to as "adjustments [14]" by chiropractors or "manipulation [13]" by other providers. HVLA-SM is typically associated with a cavitation sound produced when the synovial joint linings are quickly separated. In LVVA-SM the loads are applied slowly, cyclically, and the amplitude of each load may vary.

The general theory is that the mechanism of action of SM may be related to the impact that manipulative forces have on tissues surrounding the low back. At the same time, this study is considering the nature of LBP itself. Could SM produce its beneficial effects by altering some deficit in the spinal structure or function?

### Sensorimotor Function Tests

It is well known that the spine is intrinsically dependent on stabilizing muscle forces. Crisco and Panjabi demonstrated in cadaveric studies that the ligamentous spine can sustain around 88 N of compressive load before buckling [15-19]. Therefore, neuromuscular control and coordination are important for the normal postural stability and daily movements of the spine [15-17,20-22]. Additionally well-coordinated muscle contractions can prevent overloading ligaments and joint capsules beyond their physiological limits [15,16,18,19]. There is increasing evidence suggesting that the dysfunction of muscle control and coordination (i.e., sensorimotor function) might render the spine unstable or prone to injury [15,16,18,19,23-33].

Muscle control depends on input from length and tension receptors in muscles as well as other proprioceptors in and around spinal joints. Panjabi proposed a hypothesis that links damage in the spinal ligaments and discs to muscle control dysfunction seen in chronic back pain [34]. Inaccurate feedback from proprioceptors in ligaments, muscles and joints may prevent proper initiation of protective muscle responses [35]. The precise nature of the proposed sensorimotor dysfunction is still unknown. There is evidence that biomechanical function in patients with LBP is altered, as measured by tests such as postural sway, response to sudden impact loads, and repositioning accuracy [36]. All of these functional tests involve sensorimotor function to some extent.

### Linking SM to Sensorimotor Dysfunction

There is important work showing that forces of the magnitude of SM loads can stimulate proprioceptors in the joints and muscles [37,38]. The approach in this current study is to use SM as a tool to influence proprioceptive input to spinal tissues and observe the effects that input has on sensorimotor function. Thus, this study may provide clues to the sensorimotor mechanisms that underlie the observed functional deficits associated with LBP, as well as the mechanism of action of SM.

#### Postural Sway

The capability of a person to maintain balance in an upright posture requires a complex integration of accurate sensory input and precisely coordinated motor output [39]. Sensory inputs include the vestibular system, the visual system and the proprioceptors in muscles and joints. Muscle activity must be simultaneously controlled at three levels to achieve stability: spinal reflex, brain stem balance and cognitive programming [40]. Disturbances to the neuromuscular system can affect the degree of efficiency and accuracy with which posture is maintained [41].

LBP patients have impaired postural stability compared to healthy individuals [42-46]. It is hypothesized that the reduced proprioceptive acuity derived from muscle or joint mechanoreceptors can be a cause of altered postural sway [47]. Another theory is that LBP patients have impaired short-term memory that leads to delays in processing postural control information and increased sway [48].

#### Response to Sudden Impact Loads

In the flow of everyday living, people sometimes experience sudden and unanticipated forces, such as stepping off a curb unexpectedly. An individual's central nervous system is designed to deal with these challenges, or sudden loads, to the system in such a way that minimal disruption occurs to the individual's current activities. These sophisticated procedures include the rapid activation of muscles to oppose the sudden load and the concurrent relaxation of the corresponding antagonistic muscles. It has been found that LBP patients respond differently compared to healthy individuals [49-52]. It takes their muscles longer to respond and their response is smaller [53,54]. Several studies demonstrated that the response of the trunk musculature is dependent on the direction of perturbation [32,55-58]. There also appears to be a correlation between muscle control delays in the sudden load test and the increased postural sway in LBP patients [59].

#### Repositioning Accuracy

A higher-order integration of proprioception, mostly from mechanoreceptors in the skin, muscles and joints, allows the body to sense the position of its parts in space [60,61]. Mechanisms of proprioception have been extensively studied in the limbs. Limb repositioning tasks involve sense of position, memory of how a position felt and motor response to return the body part to the same position. Researchers have recently applied the repositioning approach in studying LBP and found that patients with chronic LBP had a decreased accuracy of lumbo-pelvic repositioning compared to pain-free controls [62-65]. Pain-free controls have a repositioning error between 1-2 degrees, while LBP patients have an error about twice as great, most likely due to altered proprioceptive input from the lumbar spine [47,62,64,66,67]. On the other hand, some studies did not find differences in the position sense in low back pain patients compared to healthy individuals [67-71]. It has been suggested that impaired proprioception may contribute to the poorer repositioning accuracy in patients with LBP. Brumagne et al. demonstrated that vibration of the multifidus muscle led to an increase in the repositioning error in pain-free participants, providing evidence that muscle spindles are an important element of lumbar proprioceptive ability [72]. Conversely, muscle vibration in LBP patients decreased the repositioning error. This suggests that LBP and pain-free individuals are different in the way they process spindle information [72]. Lee et al. suggested that it might be possible to detect proprioceptive differences between various subgroups of LBP patients using tests with a higher motion perception threshold [65].

### Aims

The primary aims of this study are to 1) determine immediate pre to post changes in sensorimotor function in the lumbopelvic region as measured by postural sway following delivery of a single HVLA-SM or LVVA-SM treatment when compared to a sham treatment consisting of light effleurage and a sham mechanically assisted adjustment and 2) to determine changes from baseline to 2 weeks (4 treatments) of HVLA-SM or LVVA-SM compared to a sham treatment.

Our secondary aims are to:

a) Determine immediate pre to post changes in sensorimotor function in the lumbopelvic region as measured by response to sudden impact loads and lumbar repositioning accuracy error following delivery of a single HVLA-SM or LVVA-SM treatment when compared to a sham treatment;

b) Determine pre to post changes in sensorimotor function in the lumbopelvic region as measured by response to sudden impact loads and lumbar repositioning accuracy error following HVLA-SM or LVVA-SM when compared to a sham treatment following the delivery of 4 treatments over a 2 week period;

c) Estimate the effects of 6 weeks (13 applications) of HVLA-SM and LVVA-SM to the lumbopelvic region on the 3 measures of sensorimotor function; and

d) Explore whether changes in sensorimotor function are associated with changes in self-reported back pain intensity, disability, or health status at 2 weeks (after 4 SM visits) and at 6 weeks (after 13 SM visits).

## Methods/Design

This study was funded as a cooperative agreement with NCCAM/NIH as part of a developmental center (1U19AT004137) designed to collect preliminary data as well as increase the research expertise and infrastructure of the Palmer Center for Chiropractic Research (PCCR). The central scientific theme of the overall developmental grant is focused on SM, LBP and mechanisms of action of SM. The grant includes an NIH appointed External Advisory Committee (EAC) which meets annually to discuss the study plans and progress. The addition of the sham / "doctor's choice" treatment group was recommended by the EAC rather than the wait-list control group proposed in the original grant application. We also added exploratory aims to assess the doctor and patient perception of the quality of the SM delivery. These additional aims will be discussed in future papers.

### Overview

Approximately 219 individuals with acute, subacute or chronic low back pain are being allocated in a 1:1:1 ratio to receive either 13 HVLA-SM treatments over 6 weeks to the lumbar and pelvic region, 13 LVVA-SM treatments over 6 weeks to the lumbar and pelvic region, or 2 weeks of a sham treatment followed by 4 weeks of full spine "doctor's choice" SM. Primary and secondary outcomes are assessed at baseline visit 2 (BL2), week 2 and week 6 by assessors blinded to treatment assignment.

### Study population

Participants are recruited from the general population of approximately 400,000 adults living in the Quad Cities area (QCA) of Iowa and Illinois. The QCA is a combination of 4 cities: Davenport, IA, Bettendorf, IA; Moline, IL; and Rock Island, IL. This study is being conducted at the PCCR, which is located on the Palmer College of Chiropractic (PCC) main campus in Davenport, IA.

The study protocols and the informed consent documents have been approved by the PCC's institutional review board (IRB) (PCC IRB# 2007M093). A Data and Safety Monitoring Committee is also overseeing study progress.

### Inclusion and exclusion criteria

The inclusion and exclusion criteria are listed in Table 1. Volunteers between the ages of 21 and 65 years with acute, sub-acute or chronic low back pain who are willing and able to sign informed consent documents are eligible for this study. Participants must also have a numerical pain rating scale (NRS) greater than or equal to 4 (on a scale of 0 - 10) at either the initial computer assisted telephone interview (CATI ) or the baseline 1 in-person interview. In addition, NRS measurements cannot be less than 2 at any screening visit.

**Table 1:** Inclusion and Exclusion Criteria

Inclusion criteria	Rationale	Source
Age 21 y -65 y inclusive	Individuals > 65 are not as likely to tolerate the biomechanical tests and experience altered sensorimotor function. Children not considered for study	PS, BL1 Int

Acute, sub-acute or chronic LBP matching QTF classifications 1, 2, 3 or 7	Low back pain, uncomplicated by known nerve compression, neurological signs or prior surgery	Exam, CR

Numerical Pain Rating Scale of > 4 at PS or BL1 **&**≥2 at PS, BL1, BL2	Low back pain with enough severity to demonstrate clinical improvement	PS, HA1, HA2

Signed the Informed Consent Documents	Research policy	PS, BL1 Int, BL2 Int

**Exclusion criteria**	**Rationale**	**Source**

Bleeding disorders	Safety concern for biomechanical testing procedures	Exam, CR

Sensitivity to tape used during the sensorimotor function tests	Safety concern for biomechanical testing procedures	BL1 Int, Exam

Pregnancy or nursing	Safety concern for biomechanical testing procedures	PS, BL1 Int, BL2 Int

Contra-indication to SM	Safety concern for treatment protocols	Exam, CR

Joint Replacement	Safety not confirmed for biomechanical testing	PS, BL1 Int, Exam, BL2 Int

Pacemaker	Safety not confirmed when used near biomechanical equipment producing electromagnetic field	PS, BL1 Int, Exam, BL2 Int

Extreme obesity (≥ 307 lbs)	Safety concern related to equipment weight capacity	BL1 Int

Vascular claudication	Condition can result in intolerance to biomechanical testing protocols	PS, Exam, CR

Bone and joint abnormality	Condition can result in intolerance to biomechanical testing or treatment protocols	PS, Exam, CR

Inflammatory or Destructive tissue changes to the spine	Condition can result in intolerance to biomechanical testing or treatment protocols	Exam, CR

Osteoporosis	Condition can result in intolerance to biomechanical testing or treatment protocols	PS, Exam, CR

General poor health	Overall condition is too poor to tolerate both treatment and biomechanical testing procedures	Exam, CR

Neuromuscular Diseases	Condition might interfere with data collection	PS, Exam, CR

Peripheral Neuropathies	Condition might interfere with data collection	PS, Exam, CR

Spinal Surgery	Condition might interfere with data collection	PS, Exam, CR

Suspicion of drug or alcohol abuse	Condition can interfere with ability to comply with study protocol and data collection	BL1 Int, Exam, CR

Uncontrolled hypertension	Condition might make it difficult to receive treatment or perform research procedures	PS, BL1 Int, Exam

BDI-II ≥ 29	Condition can interfere with ability to comply with study protocol and data collection	HA1

QTF 4-6 & 8-11	Condition of sufficient complicated nature to cause intolerance to biomechanical testing procedures or data collection	Exam, CR

Cauda Equina Syndrome	Requires emergency surgical evaluation	Exam, CR

Inability to read or verbally comprehend English	Difficult to ensure full consent	PS, BL1 Int

Further diagnostic procedures other than dipstick urinalysis or x-rays	Advanced diagnostic testing was unavailable	Exam, CR

Retention of legal advice and open or pending case related to LBP	May interfere with study compliance	PS, BL1 Int, BL2 Int

Ongoing treatment for LBP by other health care providers	Possible confounding data	PS, BL1 Int, BL2 Int

BDI-II- Beck Depression Inventory; BL1- Baseline Visit 1; BL2- Baseline Visit 2; CR- Case Review; Exam- Determined by the examining clinician; HA1- Health Assessment Questionnaire administered at BL1; HA2- Health Assessment Questionnaire administered at BL2; Int- Study Coordinator administered Interview; PS- Phone CATI Screen; QTF- Quebec Task Force; SM- Spinal Manipulation

Exclusion criteria have been developed to screen out patients with: safety concerns for either SM or the sensorimotor testing (e.g. bleeding disorders, sensitivity to tape, contra-indication to SM, pregnancy); unconfirmed safety (e.g. joint replacement, pacemaker); safety concern related to equipment weight capacity (e.g. extreme obesity with weight greater than or equal to 307 pounds); conditions that may result in intolerance to biomechanical testing or treatment protocols (e.g. vascular claudication, bone and joint abnormality, inflammatory or destructive tissue changes to the spine, osteoporosis, Quebec Task Force classification 4, 5, 6, 8, 9, 10, 11 [73]); overall condition too poor to tolerate treatment and biomechanical testing procedures; conditions that may interfere with data collection (e.g. neuromuscular diseases, peripheral neuropathies, prior spinal surgery); conditions that might interfere with data collection and ability to comply with study protocol (e.g. suspicion of drug or alcohol abuse, uncontrolled hypertension, depression according to the Beck Depression Inventory-II^©^); condition which requires surgical evaluation (e.g. cauda equina syndrome); and other concerns which may make it difficult to fully consent, interfere with study compliance, or constitute a possible data confounder (e.g. inability to read or verbally comprehend English), indicators for diagnostic procedures beyond dipstick urinalysis or x-rays, retention of legal advice for an open or pending case related to LBP and ongoing treatment for LBP by other health care providers.

### Recruitment procedures

Initial recruitment efforts were jumpstarted through a well-received press release to the local media when the study began. Ongoing efforts have focused on direct mailers to all of the QCA.

### Screening and eligibility

**Figure 1:**
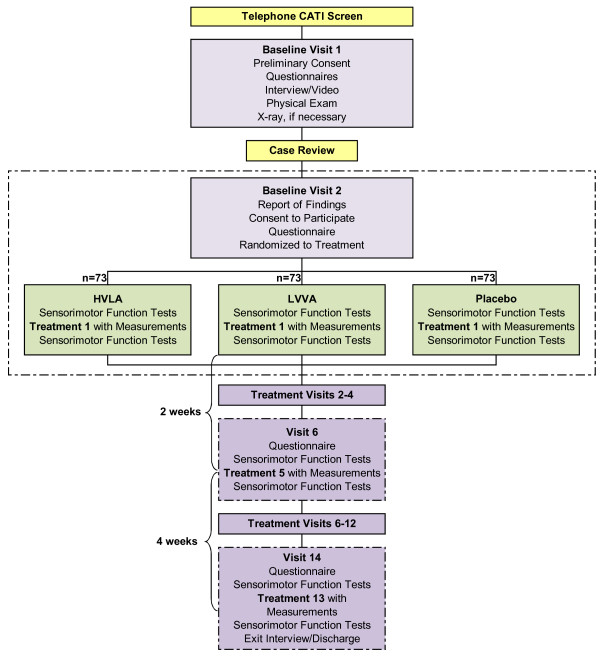
**Participant flow chart**.

Participants are screened for eligibility using a CATI and if eligible scheduled for a baseline in-person screening interview and physical examination. At this appointment, baseline visit 1 (BL1), participants first complete a HIPAA notice of privacy form, provide contact information and sign an initial informed consent document that allows us to use their baseline information even if they are not enrolled in the study. They then complete questionnaires on their past medical history and provide baseline responses to the self-report outcomes. A study coordinator obtains participants' height and weight and reviews all forms for missing or incomplete data, clarifies any questionable entries, and enters eligibility items into a web-based interface programmed with eligibility checks. If a participant is eligible for further screening, the study coordinator verbally provides an overview of the study using a flow chart (Figure 1), explains the forthcoming informed consent documents in detail, and shows a video demonstrating the biomechanical and treatment procedures used in the study.

A research clinician reviews the participant's past medical history and initiates the clinical evaluation with questions regarding relevant aspects of the health history. The clinician then obtains a focused history of the participant's low back pain and performs an eligibility examination. To assist with diagnosis, radiographs and urinary analysis may be ordered; if any other diagnostic procedure is necessary to evaluate the condition, the participant is excluded from the study and referred to an appropriate healthcare provider. When available, and with participant consent, health records are requested and reviewed as part of the screening process.

#### Confirmation of diagnosis and determining eligibility for the study

Following the participant examination, the clinician prepares a case summary report detailing the participant's low back complaint, past medical history, exam findings, diagnostic test results (if present), and diagnosis. Twice-weekly case review meetings with study clinicians and other clinic personnel are held to review findings for all who complete the BL1. Case review meetings are intended to: 1) facilitate consistent interpretation and application of pre-defined eligibility criteria; 2) utilize the combined clinical experience of multiple clinicians; 3) ensure safety; and 4) reach an evidence-based consensus of diagnosis for each participant.

A study coordinator prepares the case review agenda and ensures clinic charts and other related records are available for case review meetings. The project manager prepares electronic presentation of the case summary and digital imaging (if present). All electronic documents and images containing personal health information are stored in a secure, password-protected network file server.

Case review meetings encompass a verbal presentation of each case by the clinician who performed the exam. After presentation, the clinician allows panel members to electronically review the report and ask questions. The clinician then leads the team through several key discussion points including x-ray review (if present), compliance, safety, diagnosis and eligibility determination. An electronic manual of operating procedures is available to reference eligibility criteria and operational definitions as they apply to decisions made at this meeting. Any recommendations for further evaluation or referral are also determined by the panel. The group then determines the participant's eligibility by a final review of the exclusion criteria and the presenting clinician's recommendation. If consensus (80% of the present clinicians) cannot be reached, the senior clinician makes final participant eligibility determination. Dissenting clinical opinions are recorded. Participants excluded at case review are contacted by the examining research clinician who informs them that they are ineligible for the study, reports their exam findings and provides recommendations for follow-up with non-study providers.

#### Baseline Visit 2

Treatment allocation occurs at BL2. First, a clinician provides the participant with a verbal report of the BL1 exam findings, probing for any clinically important change in the participant's condition or new issues that may impact study compliance, and answering any questions the participant has regarding their condition or study participation. The study coordinator then meets with the participant and answers any questions about the study, provides an opportunity for the participant to view the video showing the biomechanical tests and treatment procedures again, and probes for potential compliance issues. For eligible participants, the study coordinator obtains and witnesses the participant's signature on the final informed consent document. Next, the participant completes a questionnaire to ensure that their NRS score is not less than 2. Finally, the study coordinator confirms eligibility and allocates the participant to one of the three treatment groups.

#### Treatment Allocation

A web programmer wrote the code and a data manager validated the code for an adaptive computer-generated treatment allocation algorithm based on the minimization method of Taves to allocate participants to one of three treatment groups in a 1:1:1 allocation ratio [74]. The minimization algorithm balances group differences over 3 baseline factors: age (21-39; 40-49; 50-65), sex and duration of pain ( < 4 weeks; 4-12 weeks; 12 weeks- < 6 months; 6 months- ≤ 1 year; > 1 year). The study coordinator selects the participant unique identification (ID) number from a dropdown list in a web-based interface that produces the coded treatment group allocation. The date and time of allocation, study personnel user ID and group assignment are stored in the project database. All study personnel are blinded to upcoming treatment allocations and the biomechanical examiners remain blinded to treatment group throughout the study. If the web system becomes unavailable due to server failure, a back-up treatment allocation protocol is administered with predetermined sequentially numbered, opaque envelopes stored in a locked cabinet in a secure area. Subsequent allocations via the minimization algorithm take into account all participants' minimization variables.

### Study Treatments

#### HVLA-SM

HVLA-SM is performed with the participant in the lateral recumbent or side-lying position. Participants attain a side-lying position with the free hip and knee slightly flexed and adducted while the lumbar spine and pelvis remain roughly perpendicular to the treatment surface. The hip and knee on the weight bearing side are extended or very slightly flexed. The doctor stands in front of (facing) the participant while stabilizing the free thigh and leg with their own thigh. The participant's shoulder is stabilized with the doctor's hand (stabilizing hand) while the participant's forearms rest across the chest or abdomen. A high-velocity low-amplitude manipulative thrust is applied with the doctor's other hand (thrust or contact hand) on specific areas of the participant's lumbar vertebrae (mamillary process, spinous process) or pelvis (posterior superior iliac spine, ischial tuberosity, sacral ala, 1^st ^sacral segment, 3-4^th ^sacral segment), depending on the condition, physical findings and treatment objective. Short, controlled movement of the doctor's upper body, shoulder and arm, often combined with a slight falling or "body-drop" movement creates the motion, momentum and position for a HVLA-SM thrust, which is delivered through the contact hand [75]. The thrust vector varies with therapeutic intent and point of contact [13,76]. The doctor does not thrust with the stabilizing hand; however, to maintain participant stability on the treatment table, mild counter-pressure is frequently necessary.

#### LVVA-SM

This technique has been used at the Palmer Center for Chiropractic Research in previous studies [75]. In brief, participants lie face down on a specially designed table that allows the doctor to apply a controlled motion to the participant's lumbopelvic region [75,77]. The doctor stands to one side and forms a manual contact with the spinous process of a lumbar vertebra or on the ilium. This contact resists the distractive force created by flexing and distracting the table supporting the lower extremities, which is controlled by the doctor's other hand. This procedure allows for a relatively focused distractive force under the manual vertebral contact and is combined with flexion, lateral flexion or circumduction movements generated from moving the table beneath the lower extremities. Individual motions are determined based on characteristics of the condition and therapeutic aim. Distraction combined with specific movements occurs in 1-3 second intervals, in sets of 5-20. One to three sets are typically delivered in a given lumbar or sacroiliac area. Participants are constantly monitored for tolerance to the amount of motion and distractive force used during the procedure.

#### Sham Treatment

The sham treatment protocol, developed by the investigative team, is composed of two parts. The first part includes three light effleurage strokes, or patterns, applied with no greater than 30 N of pressure [78]. First, the basic heart shaped stroke is performed three times, followed by three "L" shaped strokes on each side of the thoracic and lumbar areas. Three to four tree strokes follow, ending with heart shaped strokes. The procedure spans three minutes +/- 15 seconds. The second part of the sham protocol includes the use of an Activator IV device. An Activator IV (Activator Methods International, Ltd, Phoenix, AZ) is a handheld spring-loaded mechanical device used to deliver a high-velocity low-amplitude thrust and is commonly used within the chiropractic profession. For use as a sham treatment, a plastic or rubber guard is placed on the Activator tip rendering it incapable of producing a thrust. As is common in conjunction with the use of this device, the clinician evaluates the lumbo-pelvic region by lightly palpating the iliac crest and lower lumbar vertebrae and also examines the length of the participant's legs by gently pushing their feet together. The clinician then places their thumb on a lumbar spinous process and again in the sacroiliac region while simultaneously triggering the Activator so that a "click" is heard. Though incapable of producing a thrust, the device still produces the same sound.

### Sensorimotor Function Tests

**Figure 2:**
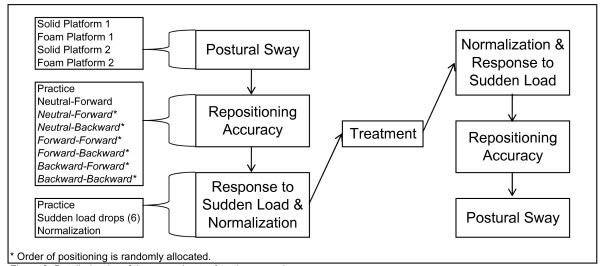
**Detailed order of the sensorimotor function procedures**.

The sensorimotor function tests applied in this study include postural sway, lumbar repositioning accuracy, and response to sudden load. The participant performs one set of these tests immediately before the treatment and another set immediately after the treatment at the BL2, week 2 and week 6 visits (up to six sets of sensorimotor function tests in total). The detailed order of the tests is illustrated in Figure 2.

#### Postural Sway

The ability to maintain balance in an upright standing posture is monitored using a force plate (Model # 4060-NC, Bertec, Inc, Columbus OH), which measures the posture sway (i.e., the movement of the center of pressure) in the anterior-posterior (X) and side-to-side (Y) directions. The participant stands quietly on either a solid platform (i.e., directly on the force plate) or a soft surface (i.e., on a 10 cm thick latex foam pad) for a period of 35 seconds while blindfolded and wearing socks without shoes. The first 30 seconds of data are recorded at a sample rate of 1000 Hz using a Motion Monitor data acquisition software (Innovative Sports Training, Inc., Chicago, IL). Three postural sway outcomes are extracted including the mean sways in the X and Y directions away from the center and the mean planar sway speed.

To keep the postural sway test consistent through the study, the participant is instructed to stand in their neutral stance. The biomechanical examiner then places the participant's feet next to one of the four preset stance widths (24, 30, 36, or 44 cm apart between the lateral malleoli, respectively) that is the closest to the neutral stance. The same stance width is applied in the subsequent tests. However, the participant is allowed to choose their own foot flare angles for each set of sensorimotor function tests. The flare angles may be different for the solid platform and the foam platform [79,80].

#### Repositioning Accuracy

The lumbar repositioning accuracy, or the ability to return to the target position (i.e., the initial lumbar curvature) following a series of pelvic movements, is monitored using a Polhemus electromagnetic motion capture system (Polhemus Liberty, Polhemus, Colchester, VT). Two Polhemus position tracking sensors are attached to the back of the participant at the T12/L1 and S1 levels, respectively, using double-sided adhesive foam pads. The curvature of the lumbar spine is defined as the inclination difference between the two sensors. The changes in lumbar curvature are recorded using a Motion Monitor data acquisition workstation at a sampling rate of 120 Hz. To ensure the sensor placement consistently through the study, the heights of the sensors from the seating platform are recorded and applied in the subsequent tests.

For this test, the participant sits on a rocking seat with a 12 cm diameter cylindrical base that allows for forward and backward tilting, but no sideways tilt. The feet are kept flat on the floor and the chair elevation is set so that the thighs are horizontal. The rocking motion is centered in the pelvis and participants are instructed to keep their head and upper trunk vertical. Participants are blindfolded throughout the procedure to eliminate visual cues of position. Each test starts in one of seven motion configurations under the guidance of the biomechanical examiner. Participants learn the target position by holding the posture for 5 seconds. On a cue from the examiner, the participant moves their pelvis back and forth in a rocking motion while keeping their head and upper torso upright. At the end of 5 full cycles of motion, the participant is instructed to return to their target position and hold the posture for 5 seconds. The 3 target positions are neutral, forward, and backward sitting postures. The first motion is always the neutral starting position followed by a forward motion. Subsequently, there are two motion directions, either flexion or extension within each target position. Therefore, in addition to the first standardized motion, there are six additional combinations of motion configurations. The sequence of these motions is assigned by predetermined random permuted blocks stored in the web system. Figure 2 demonstrates one of the testing sequences.

To prevent aggravating the participant's back pain, as well as allowing the participant to get familiar with the procedure, the biomechanical examiner first carefully guides the participant through one mock repositioning test. If an increase in pain is reported in either the backward or forward direction, the corresponding target positions are eliminated (e.g. dropping to four target positions). The participant is also instructed to move within their comfortable range. If the pain increases in both directions, the whole lumbar repositioning test is eliminated. Additionally, if there is an increase in pain reported later in the test, the same caution is applied.

#### Response to Sudden Load

The response to sudden load, as characterized by the paraspinal muscle response time and activity level following an unexpected pull at the upper chest, is monitored using a Delsys Bagnoli EMG System (Delsys Inc, Boston, MA) when the participant stands in the exact same posture as that of the postural sway on the hard surface. EMG electrodes are attached to the paraspinal muscles bilaterally 3 cm from midline at the L3 level. Six sudden loads with random rest periods in between are applied such that the participant cannot expect the incoming loads. Additionally the participant is blindfolded and listening to white noise to block their visual and hearing cues. Four seconds of data around the sudden loading events are recorded using the Motion Monitor data acquisition software at a sampling rate of 1000 Hz. To prevent aggravating back pain, as well as allowing the participant to get familiar to the procedure, the biomechanical examiner first carefully guides the participant through one mock drop test without blocking their vision and hearing. If an increase in pain is reported, the procedure, as well as EMG normalization, is discontinued for that visit.

The sudden load applied at the chest is achieved using a light weight (1.6 kg) dropping at a preset height. The preset drop height is calculated based on the participants' height and weight (Drop Height (cm) = 6.582 + 0.0971 * weight (kg) + 0.0854 * height (cm)). A stiff sensor bar is held tightly to the participant's chest at the level of the manubrium (just above the breasts) using nylon straps around the back. A load cell (Model # LC101-100, OMEGADYNE Inc., Sunbury, OH) and an accelerometer (Model # CXL10LP3, Crossbow Technology Inc., Milpitas, CA) are rigidly attached to the sensor bar. A rope, passing over a pulley, connects the drop-weight to the force transducer.

The readings at the load cell are used as a trigger to start the data acquisition system. To keep the sudden load test consistent through the study, the height of the rope attachment at the chest is measured from the floor and used in the subsequent tests. The outcome variables to be extracted from the EMG data include the response starting time (i.e., the delay between the rising in EMG activity and the rising in load cell readings), the peak response time and the peak magnitude from the right and left paraspinal muscles, respectively.

To enable comparison between participants, an EMG normalization procedure is performed. The participant lays face down on a bench, with upper trunk extending out over the front edge and supported by the elbows. The legs are strapped to the table at the buttocks and at the ankles. EMG is recorded when the participant lifts their elbows off the support using their back muscles and holds the posture for 5 seconds. EMG is also recorded for 5 seconds before the lifting and for another 5 seconds after the participant places their elbows back to the support. If the participant reports an increase in pain during the pre-treatment lifting phase, the normalization procedure is discontinued. In that case, the post-treatment normalization and sudden load tests are also not conducted. If there is no increase in pain before the treatment, but there is during the post-treatment normalization lifting phase, both the post-treatment normalization and sudden load tests are discontinued.

### Self-report outcome assessments

In addition to sensorimotor function, we are also collecting data on sociodemographic characteristics, back pain history, potential confounders, and back pain outcome measures (Table 2). The data are collected at BL1, BL2, week 2 and week 6.

**Table 2:** Baseline and follow-up assessments

Measures	Baseline 1	Baseline 2	Week 2	Week 6
**Baseline Information**				

Sociodemographic characteristics	X			

Back pain history	X			

**Sensorimotor Functions Measurements**				

Postural Sway		Pre & Post Treatment	Pre & Post Treatment	Pre & Post Treatmentt

Response to Sudden Impact Loads		Pre & Post Treatment	Pre & Post Treatment	Pre & Post Treatment

Lumbar Repositioning Accuracy		Pre & Post Treatment	Pre & Post Treatment	Pre & Post Treatment

**Self-report Outcome Assessments**				

Roland Morris Disability Questionnaire (RMDQ)	X		X	X

Numerical Pain Rating Scale (NRS)	X	X	X	X

Quality of Life (SF-36)	X			X

Beck Depression Inventory (BDI-II)	X			X

Bothersomeness of low back pain	X	X	X	X

Low Back Pain Definition	X			

Fear Avoidance Beliefs Questionnaire	X			X

Satisfaction with back care				X

Treatment-Related Information*				

Adverse experiences		X	X	X

Patient's Quality of Treatment Perception		X	X	X

Clinician's Quality of Treatment Perception		X	X	X

**Assessment of Potential Confounders**				

Use of co-intervention: medications*	X	X	X	X

Use of co-intervention: manual therapy*	X	X	X	X

Exercise and job work load	X			

Smoking status	X			

Body Mass Index	X			

*-Also occurs at all treatment visits

The modified *Roland-Morris Disability Questionnaire (RMDQ) *24 item version assesses LBP-related disability. The RMDQ may be the most common and respected LBP assessment instrument in LBP outcome research [81]. Clinical improvement over time is graded based on the analysis of serial questionnaire scores. The minimum clinically important difference (MCID) is estimated at 2 points [82]. The RMDQ is a one-page questionnaire related to LBP disability with documented reliability and validity [83] that has been shown to be sensitive to clinical change in patients with low back pain [84-86].

Participants are asked to rate their level of pain on an ordinal 11-box scale (0 = no LBP; 10 = worst LBP possible) at baseline and before each treatment. The *numerical pain rating scale (NRS) *has excellent metric properties, is easy to administer and score, and has received much use in LBP research [87]. The question captures information pertaining to pain over the past 24 hours. The MCID is a change of 2.5 points [87].

The *bothersomeness of symptoms *commonly associated with LBP is measured using an existing instrument from the LBP literature. We ask volunteers to rate the bothersomeness of their LBP are during the past week, measured on a 1 to 5 scale (1 = not at all bothersome and 5 = extremely bothersome). Bothersomeness questions are practical and have demonstrated good internal consistency, construct validity, and responsiveness to change with time in patients with LBP and sciatica [88].

The *Medical Outcomes Study 36-Item Short-Form Health Survey *version 2 (SF-36v2) is used for characterizing the physical and mental health of our patients. It has been used extensively in LBP research [89,90]. The metric properties of the SF-36 as an outcome instrument have been exhaustively studied with generally excellent results [91]. For the purposes of this study we are interested in looking at physical function and bodily pain. The MCID for physical function is 1 point and bodily pain is 4 points [92].

### Data collection and management

Information is collected at every stage of recruitment, treatment allocation, and throughout treatment, so that the patient flow can be reported according to the CONSORT guidelines [93]. Specifically, we collect recruitment source, total number of responses per recruitment source, potential participants' resolution (e.g. ineligible, do not wish to participate, allocated), the number allocated to each treatment group, participant compliance to treatment protocol, the number lost to follow-up, and the number of participants completing the trial.

Participant self-report questionnaires are collected on paper forms. The only identifier on a given data collection form is a unique participant ID; no other personal identifiers are recorded on these forms. Study coordinators have oversight for all paper data collection forms, log each completed form into a form tracking interface of the web system and submit data forms for key-entry weekly. Paper data collection forms are stored in locked filing cabinets. The paper forms are double key-entered by trained data entry clerks in an MS Windows program using range and validation checks to improve accuracy.

The project's web system is password-protected and uses a Microsoft SQL Server database platform to store all data. Study personnel have unique user IDs and passwords restricting access from a Main Menu. All data collected by study personnel are recorded in user-friendly data-entry interfaces. Study coordinators assure that clinicians and biomechanical examiners complete paper data collection and web data-entry for the chiropractic research measures. The data manager creates the data dictionaries and datasets for analysis.

Quality control procedures are utilized to ensure that recruitment is on schedule, treatment allocation is occurring as planned, data collection protocols are being used accurately, data collected through the CATI and other web interfaces are being stored correctly in the SQL databases, sensorimotor outcome variables are extracted from the biomechanical measurements within 2 weeks of data collection and that the data are being transferred and retrieved properly.

### Protection of human subjects and assessment of safety

This study was reviewed by the PCC's IRB (#2007M093). In addition, all adverse events in the trial are summarized in routine reports submitted to the IRB quarterly.

This trial is monitored by a Data and Safety Monitoring Committee (DSMC) comprised of a biostatistician, medical physician, doctor of chiropractic and epidemiologists/clinical trialists, none of whom are affiliated with Palmer. The DSMC's responsibilities are to ensure the overall safety of the study participants and provide the principal investigator with advice about the scientific and ethical conduct of the study. The study biostatistician prepares a study report for the DSMC including accrual plots and other enrollment data, data collection forms processing status, baseline characteristics of enrolled participants, follow-up and treatment compliance, protocol violations and all web-based reportable adverse events (see *Adverse Events) *every 6 months. The DSMC meets in person annually and by teleconference as needed.

### Safety Monitoring

All participants involved in the study are monitored by licensed doctors of chiropractic serving as research clinicians. Research clinicians routinely evaluate participants for status change by probing for adverse events and performing clinical evaluations at each visit throughout the study period.

#### Adverse Events

We monitor safety at two levels: 1) adverse events (AE) that are possibly, probably, or definitely related to the practice of spinal manipulation and 2) serious adverse events (SAE) regardless of their attribution. For this study, we define an AE as any untoward medical occurrence that may present itself during the conduct of the study and which may or may not have a causal relationship with the study procedures. The Adverse Event Grading and Reporting Protocol defines when and how these events are reported to the DSMC and the IRB. Clinicians assess whether an AE is: 1) mild, moderate, severe, or serious; 2) expected (disclosed in the Consent Form or part of an underlying disease) or unexpected (more serious than expected, or not disclosed in the Consent Form); and 3) definitely related to intervention, probably related, possibly related, unlikely related or unrelated.

We use the FDA definition of SAE, which is any adverse experience occurring during treatment that results in any of the following outcomes: death, a life-threatening adverse experience, hospitalization or prolongation of existing hospitalization, a persistent or significant disability/incapacity, or a congenital anomaly/birth defect resulting from a pregnancy (21CFR314.80, revised 01 April 2010). AE information is collected during the treatment encounters and at all follow-up assessments. Participants also are instructed to contact investigators in the event of clinically important pain, discomfort or distress that they believe may be associated with treatment. We developed an automated web-based system for recording and monitoring all AE's that are possibly, probably or definitely related to study participation and all SAE's. The clinical team records unrelated and unlikely related AE's and reports these events to the IRB and DSMC quarterly. The project managers and senior clinician are responsible for tracking and reporting all adverse events to the principal investigator. The Adverse Event Grading and Reporting Protocol determines when and how these events are reported to the DSMC and the IRB when appropriate.

### Sample size justification

We calculated power using Proc Power in SAS for a total sample size of 219 by considering 63 patients per group and inflating that by 15% (73 per group) to account for drop outs. Because we anticipate no drop outs during BL2, we considered 73 per group in the power analyses for the immediate changes in the pre to post treatment measurements at the first treatment, but 63 per group for the changes in the pre-treatment variables at BL2 and week 2.

We conducted power analyses for the 3 standing postural sway characteristics, mean sway away from the center in the X and Y directions and the mean planar sway speed. These are the primary response variables and we estimated power using changes in similar variables collected from participants standing on a solid force plate, described previously, in the literature and standard deviations from our preliminary studies [47,94]. Table 3 presents a variety of power values for these variables.

**Table 3:** Statistical power based on one-way ANOVA for the 3 primary response variables testing at 0.05 level of significance using SDs from preliminary data and the literature

		Power A	Power B
		
Response Variable		Contrasts*	Contrasts*
Sway Speed	Solid	>99%	>99%
	
	Foam	86	80

Mean Sway X	Solid	76-94	69-90
	
	Foam	64-73	58-67

Mean Sway Y	Solid	88-95	83-92
	
	Foam	21-34	18-30

Effect sizes: Sway Speed: 4 mm/sec; Mean Sway X: 1 mm; Mean Sway Y: 0.5 mm [47,94] Power A is determined for n = 73 per group for the immediate changes in the pre- to post-treatment measurements at the first treatment. Power B is determined for n = 63 per group for the changes in the pre-treatment variables at the first treatment and at week 2.

*Two 1 degree of freedom contrasts to test mean differences of HVLA-SM vs Sham Control and LVVA-SM vs Sham Control.

### Statistical analysis

SAS version 9.1.3 will be used for data analysis (SAS Institute Inc., Cary, NC). Descriptive statistics of participant baseline characteristics will be presented for each treatment group to assess their comparability as well as the generalizability of the sample. We will use an intention-to-treat approach for all analyses. The analyses for the primary and secondary aims are described below. We will not impute any missing data for analysis, but will report the amount of missing data for each variable and the reason it is missing.

#### Data Analysis for Primary Aim

For each of the 3 primary response variables, the immediate changes in the pre to post treatment measurements at BL2 and the changes in the pre-treatment variables from BL2 to week 2 will be compared across the 3 treatment groups using analysis of covariance (ANCOVA) adjusting for the minimization variables. The following 2 preplanned contrasts for the 2 degrees of freedom for treatment will be tested at 0.05: HVLA-SM vs. sham control and LVVA-SM vs. sham control on the adjusted means based on the ANCOVA model. Residual plots will be used to assess the adequacy of the model assumptions. Data transformations will be explored when model assumptions are violated. Further adjustments for the following baseline characteristics will be explored to increase the precision of the estimated effects: RMDQ, NRS, Quebec Task Force classification, Beck Depression Inventory and chiropractic care (yes/no). Adjusted group means and mean differences between SM and control groups will be reported with 95% confidence intervals determined under the final ANCOVA model.

#### Data Analysis for Secondary Aim a

The remainder of the sensorimotor variables will be analyzed as above. However, only group means and mean differences between SM and control groups with 95% confidence intervals will be reported, not P-values.

#### Data Analysis for Secondary Aim b

There is no control group at 6 weeks. Therefore, changes between pre-sensorimotor variables collected at BL2 and week 6 for those allocated to the SM groups will be described separately for HVLA-SM and LVVA-SM. Means and mean differences with 95% confidence intervals will be reported for each variable.

#### Data Analysis for Secondary Aim c

Clinical outcome assessments considered include the NRS, the RMDQ and SF-36 subscales, physical function and bodily pain. Correlation coefficients and 95% confidence intervals will be reported for changes in sensorimotor response variables against changes in the outcome assessments at BL2, week 2 and week 6 time points overall and by treatment group.

## List of abbreviations

AE: adverse events; ANCOVA: analysis of covariance; AZ: Arizona; BDI-II: Beck Depression Inventory version II; BL1: baseline visit 1; BL2: baseline visit 2; CA: California; CATI: computer assisted telephone interview; cm: centimeter; CONSORT: Consolidated Standards of Reporting Trials; CR: case review; EAC: External Advisory Committee; e.g.: *exemplie gratia *or for example; DSMC: Data and Safety Monitoring Committee; EMG: Electromyography; FDA: Food and Drug Administration; HA1: Health Assessment 1; HA2: Health Assessment 2; HIPAA: Health Insurance Portability and Accountability Act; HVLA-SM: high-velocity low amplitude spinal manipulation; Hz: Hertz; IA: Iowa; i.e.: *id est *or that is; ID: identification; IL: Illinois; Int: Interview; IRB: Institutional Review Board; kg: kilogram; L3: third lumbar vertebra; LBP: low back pain; LVVA-SM: low-velocity variable-amplitude spinal manipulation; MA: Massachusetts; MN: Minnesota; MS: Microsoft; N: Newton; NC: North Carolina; NCCAM: National Center for Complementary and Alternative Medicine; NIH: National Institutes of Health; NRS: Numerical pain rating scale; OH: Ohio; PCC: Palmer college of Chiropractic; PCCR: Palmer Center for Chiropractic Research; PS: Phone Screen; QCA: Quad Cities Area; QTF: Quebec Task Force RMDQ: Roland-Morris Disability Questionnaire; S1: first sacral segment; SAE: serious adverse events; SF-36: Medical Outcomes Study 36-Item Short-Form Health Survey; SM: spinal manipulation; SQL: Structured Query Language; T1: first thoracic vertebra; VT: Vermont.

## Competing interests

The authors declare that they have no competing interests.

## Authors' contributions

DGW, WCM, RMG, CRL, EFO participated in the conception of the trial. DGW, RDV, KAP, WCM, JWD, RMG, CRL, EFO, CMG participated in the design of the trial. DGW, RDV, WCM, TX, JWD, RMG, CRL, EFO, CMG participated in plans for the analysis of the data. DGW, RDV, KAP, TX, CRL, CMG drafted the manuscript. All authors read and approved the final manuscript.
